# Activation of Muscle-Specific Kinase (MuSK) Reduces Neuromuscular Defects in the Delta7 Mouse Model of Spinal Muscular Atrophy (SMA)

**DOI:** 10.3390/ijms22158015

**Published:** 2021-07-27

**Authors:** Zhihua Feng, Steven Lam, Elena-Marie Sandino Tenn, Arundhati Sengupta Ghosh, Sarah Cantor, Wei Zhang, Pei-Fen Yen, Karen S. Chen, Steven Burden, Sergey Paushkin, Gai Ayalon, Chien-Ping Ko

**Affiliations:** 1 Section of Neurobiology, Department of Biological Sciences, University of Southern California, Los Angeles, CA 90089, USA; zfeng@usc.edu (Z.F.); stevenvlam22@gmail.com (S.L.); emstenn@gmail.com (E.-M.S.T.); buolia@gmail.com (P.-F.Y.); 2 Department of Neuroscience, Genentech, South San Francisco, CA 94080, USA; sengupta-ghosh.arundhati@gene.com; 3 Department of Neuroscience, Skirball Institute of Biomolecular Medicine, New York University, New York City, NY 10016, USA; sarahrcantor@gmail.com (S.C.); wei1868@gmail.com (W.Z.); steve.burden@nyulangone.org (S.B.); 4 Spinal Muscular Atrophy Foundation, P.O. Box 9214, Jackson, WY 83002, USA; kchen@smafoundation.org (K.S.C.); sergey@skyhawktx.com (S.P.); 5 Ultragenyx Pharmaceutical, Novato, CA 94949, USA; gai_ayalon@yahoo.com

**Keywords:** spinal muscular atrophy, neuromuscular junction, muscle-specific kinase (MuSK), skeletal muscle, innervation, denervation

## Abstract

Spinal muscular atrophy (SMA) is a motor neuron disease caused by insufficient levels of the survival motor neuron (SMN) protein. One of the most prominent pathological characteristics of SMA involves defects of the neuromuscular junction (NMJ), such as denervation and reduced clustering of acetylcholine receptors (AChRs). Recent studies suggest that upregulation of agrin, a crucial NMJ organizer promoting AChR clustering, can improve NMJ innervation and reduce muscle atrophy in the delta7 mouse model of SMA. To test whether the muscle-specific kinase (MuSK), part of the agrin receptor complex, also plays a beneficial role in SMA, we treated the delta7 SMA mice with an agonist antibody to MuSK. MuSK agonist antibody #13, which binds to the NMJ, significantly improved innervation and synaptic efficacy in denervation-vulnerable muscles. MuSK agonist antibody #13 also significantly increased the muscle cross-sectional area and myofiber numbers in these denervation-vulnerable muscles but not in denervation-resistant muscles. Although MuSK agonist antibody #13 did not affect the body weight, our study suggests that preservation of NMJ innervation by the activation of MuSK may serve as a complementary therapy to SMN-enhancing drugs to maximize the therapeutic effectiveness for all types of SMA patients.

## 1. Introduction

Spinal muscular atrophy (SMA), the leading genetic cause of infant mortality when untreated, is a motor neuron disorder caused by deletions or mutations of the survival motor neuron 1 (SMN1) gene [1,2]. The human genome also contains one or more copies of a nearly identical SMN2 gene, which differs from SMN1 in a C to T transition at position 6 of exon 7, altering splicing and leading to exclusion of sequences encoded by exon 7. The SMN2 protein is less stable and expressed at ~10-fold lower levels than SMN1 protein [3,4]. The copy number of SMN2 is negatively correlated with disease severity and responsible for different SMA subtypes [5], consistent with the idea that SMA is caused by insufficient levels of SMN protein.

SMA is characterized primarily by spinal motoneuron loss, synaptic defects, and skeletal muscle atrophy [6,7]. Dysfunction of the neuromuscular junction (NMJ) is one of the earliest and most prominent pathological hallmarks of SMA seen in patients and various animal models [8,9]. The NMJ defects include neurofilament accumulation, reduced transmitter release and active zone number, altered Ca^2+^ homeostasis, delayed maturation of NMJs and reduced acetylcholine receptor (AChR) clustering [10,11,12,13,14,15,16,17,18,19,20,21]. Furthermore, retraction of nerve terminals is observed in some vulnerable muscles resulting in severe muscle atrophy [15,18,22]. Thus, one potential therapeutic strategy for mitigating the SMA pathology is to ameliorate these NMJ defects, particularly by promoting postsynaptic differentiation and attachment of motor nerve terminals to muscle.

Clustering of AChRs on the postsynaptic muscle membranes plays a key role in synaptic function, formation, and maintenance of the NMJ. It is well established that AChR clustering is governed by the signaling pathways involving agrin and its receptor complex, including muscle-specific kinase (MuSK) and low-density lipoprotein receptor-related protein 4 (LRP4) [23,24,25,26], as well as a downstream modifier, DOK7 [27]. Studies have shown that Z+ agrin (active neuronal isoform) is poorly expressed in spinal motor neurons of SMA mice [28]. Furthermore, upregulation of Z+ agrin by a genetic approach [29] or by a pharmacological treatment [30] increases NMJ area, muscle fiber size, and innervation at NMJs of SMA mice. Similarly, enhancing DOK7 by AAV9 delivery also improved NMJ architecture and reduced muscle atrophy [31]. These findings suggest that NMJ defects can be alleviated by enhancing postsynaptic differentiation in SMA mice.

In addition to agrin, MuSK is also essential for NMJ development and maintenance [32]. Thus, enhancing MuSK activity may mitigate NMJ defects in diseases of the NMJ, such as amyotrophic lateral sclerosis (ALS) and SMA. Indeed, increasing MuSK activity either by genetic expression [33] or by a MuSK agonist antibody #13 (developed by Genentech [34]) can preserve NMJs in the SOD1-G93A mouse model of ALS [35,36]. The present study was designed to test whether stimulating MuSK activity with MuSK agonist antibody #13 can also preserve NMJs in SMA mice.

Our previous studies on the delta7 mouse model of SMA mice (a model for the severe type of SMA) have demonstrated severe NMJ denervation in a group of clinically relevant neck muscles, such as the splenius and the longissimus muscles [18]. Due to their severe denervation and high sensitivity to drug treatments, these neck muscles have been useful in analyzing NMJ deficits and the in vivo testing of various compounds, including the FDA-approved Risdiplam [37] and other SMN modifiers (e.g., [38,39]). The present work demonstrated that stimulating MuSK reduces NMJ denervation, accompanied by improved synaptic efficacy and muscle size of these vulnerable muscles in SMA mice. Although MuSK agonist antibody #13 did not increase body weight of SMA mice, its beneficial effects in promoting synapse maintenance and preventing synapse disruption may be used as a complementary therapeutic intervention for SMA patients who may be poor responders to the three currently FDA-approved SMN-enhancing drugs, Spinraza, Zolgensma, and Evrysdi [40,41,42].

## 2. Results

### 2.1. MuSK Agonist Antibody #13 Recognizes the Neuromuscular Junction

**Figure 1:**
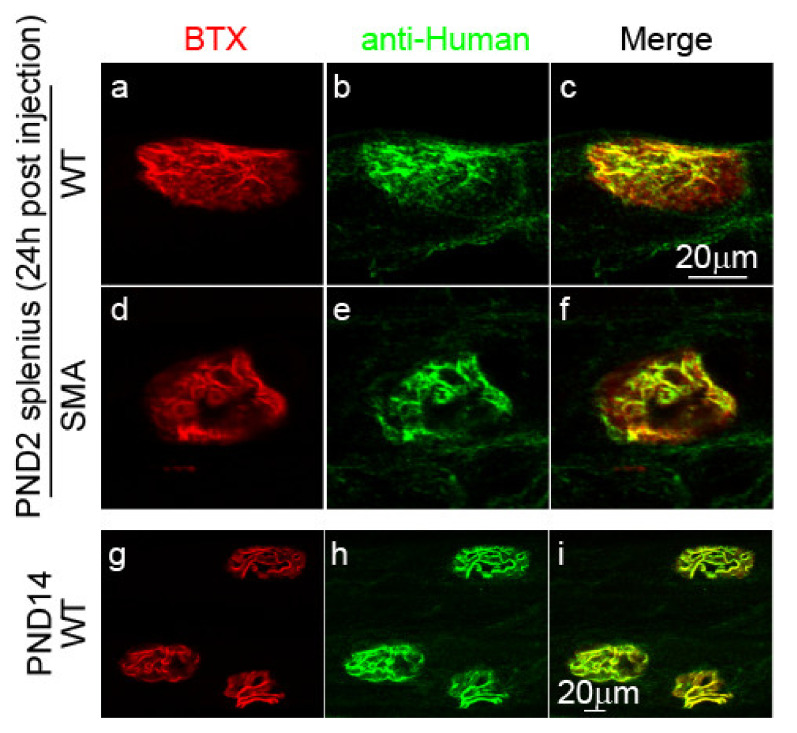
MuSK agonist antibody #13 binds to NMJs after injection. Images show PND2 WT (**a**–**c**) and SMA (**d**–**f**) NMJs labeled with BTX and anti-human secondary antibodies 24 h post-injection, and PND14 WT NMJs (**g**–**i**) after one injection at PND1 and one injection at PND9.

Before testing the effects of MuSK agonist antibody #13, we asked whether this antibody indeed binds to the postsynaptic sites where MuSK resides. To address this question, we intraperitoneally (IP) injected MuSK agonist antibody #13 (10 mg/kg) in wildtype (WT) or delta7 mice at postnatal day (PND) 2. Approximately 24 h after injection, mice were sacrificed, and the binding of MuSK antibody #13 was detected using fluorochrome-conjugated anti-human antibody. As shown in Figure 1, NMJs of the splenius muscle in both WT (a–c) and delta7 mice (d–f) at PND2 were labeled by the MuSK agonist antibody #13 (green), which is co-localized with AChRs, and stained with α-bungarotoxin (BTX) (red). Furthermore, the antibody staining persisted throughout our studies ending on PND14, as shown in an example of WT NMJs at PND 14 (g–i). These results confirmed that MuSK agonist antibody #13 binds to MuSK on the postsynaptic site of the NMJ and are consistent with the long half-life (12 days after a single injection) of the injected antibody in the blood as described previously [35].

### 2.2. The Effect of MuSK Agonist Antibody #13 on Innervation Patterns in the SMA Mice

**Figure 2:**
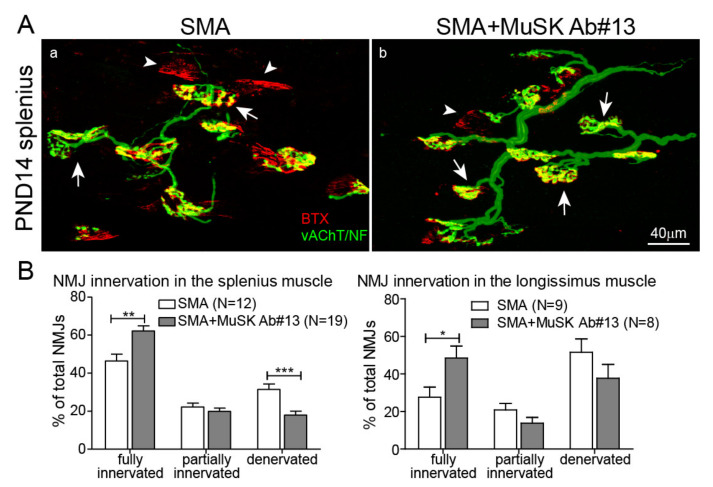
MuSK agonist antibody#13 reduces NMJ denervation. (**A**) Images show NMJs in SMA splenius muscles without (**a**) and with (**b**) MuSK antibody #13 treatments at PND14. NMJs were labeled with BTX (red), antibodies to vesicular acetylcholine transporter (vAChT) and antibodies to neurofilament (green). Arrows label fully innervated NMJs and arrowheads label denervated NMJs. (**B**) Quantitation of innervation demonstrates that MuSK antibody #13 increases the percentage of fully innervated NMJs in the splenius and longissimus muscles of PND14 SMA mice. Values are expressed as mean ± SEM, Unpaired *t*-test, * *p* < 0.05, ** *p* < 0.01 and *** *p* < 0.001.

As MuSK agonist antibody #13 binds to NMJs, we next asked whether the antibody could preserve NMJs in delta7 SMA mice, as previously shown in the ALS mouse model [35,36]. To address this question, MuSK agonist antibody #13 was administered to delta7 mice via IP injections at PND1 and PND9 and disease pathologies were assessed at PND14, the disease end stage of delta7 mice. Given that the half-life of MuSK agonist antibody #13 is ~12 days, and 2 mg/kg saturates MuSK at the synapse [35], we believe that the two injections at 10 mg/kg at PND1 and PND 9 should adequately saturate the binding. To reveal the innervation patterns, NMJs were labeled with BTX and with antibodies to vesicular acetylcholine transporter (vAChT) and neurofilament to visualize presynaptic nerve terminals. We first examined a group of muscles that are severely denervated in SMA mice, the so-called “vulnerable muscles,” such as the splenius capitis and the longissimus capitis muscles in the neck [18]. At PND14, NMJs were 100% fully innervated in the splenius muscle in WT mice [18]. As shown in Figure 2A, many denervated NMJs (arrowheads), which were stained with BTX (red) but lacked overlying nerve terminals (green), were seen in delta7 mice (a). In contrast, the number of denervated NMJs was reduced in delta7 mice treated with MuSK agonist antibody #13 (b). We quantitated denervation by categorizing synapses into fully innervated, partially innervated, and denervated groups (Figure 2B), which showed that MuSK antibody #13 promoted full innervation (46% without treatment vs. 62% with treatment, ** *p* < 0.01) while decreasing denervation (31% without treatment vs. 18% with treatment, *** *p* < 0.001) in the splenius muscle. In addition, MuSK antibody #13 also increased full innervation of the longissimus muscle (Figure 2B). These findings suggest that MuSK agonist antibody #13 improves innervation of NMJs in SMA mice.

### 2.3. The Effect of MuSK Agonist Antibody #13 on Synaptic Efficacy in the SMA Mice

**Figure 3:**
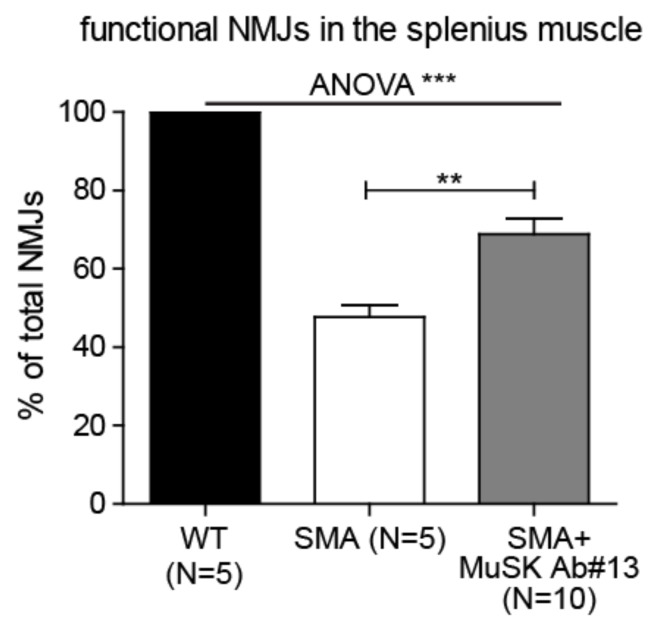
MuSK agonist antibody#13 improves synaptic efficacy in SMA mice. Endplate potentials were detected at 100% of synapses in the splenius muscle of WT, 48% of PND14 SMA mice and 69% of SMA mice treated with MuSK antibody #13. Values are expressed as mean ± SEM, ** *p* < 0.01 and *** *p* < 0.001.

To determine whether the increased innervation results in improved synaptic function, we performed intracellular recordings from NMJs in the vulnerable splenius muscle. As shown in Figure 3, evoked endplate potentials (EPPs) were detected in 100% of NMJs in WT and approximately 48% of NMJs in SMA mice. In contrast, EPPs were detected at approximately 69% of NMJs in SMA mice treated with MuSK antibody #13. These results demonstrated that MuSK antibody #13 not only improved innervation (Figure 2) but also enhanced synaptic transmission in vulnerable muscles.

### 2.4. The Effect of MuSK Agonist Antibody #13 on Skeletal Muscle Size and Fiber Number in the SMA Mice

**Figure 4:**
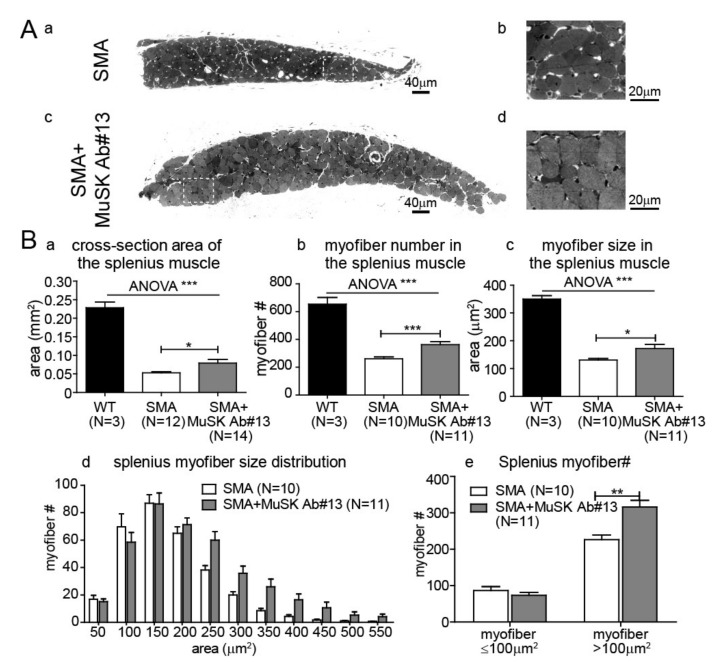
MuSK agonist antibody#13 increases the size of splenius muscle in SMA mice. (**A**) Images of cross-sections of PND14 SMA splenius muscles without (**a**,**b**)—white inset box in (**a**) is enlarged in (**b**)—and with (**c**,**d**)—white inset box in (**c**) is enlarged in (**d**)—MuSK agonist antibody #13 treatments. Scale bars: 40 µm in (**a**,**c**); 20 µm in (**b**,**d**). (**B**) Quantitation of the cross-sectional area (**a**), myofiber number (**b**), and myofiber size (**c**) in the PND 14 splenius muscle of WT, delta7 SMA mice without and with MuSK agonist antibody #13 treatments. The distributions of myofiber size (**d**) and number of myofiber less and greater than 100 µm^2^ (**e**) of PND 14 delta7 SMA mice are also shown. Values are expressed as mean ± SEM, * *p* < 0.05, ** *p* < 0.01 and *** *p* < 0.001.

**Figure 5:**
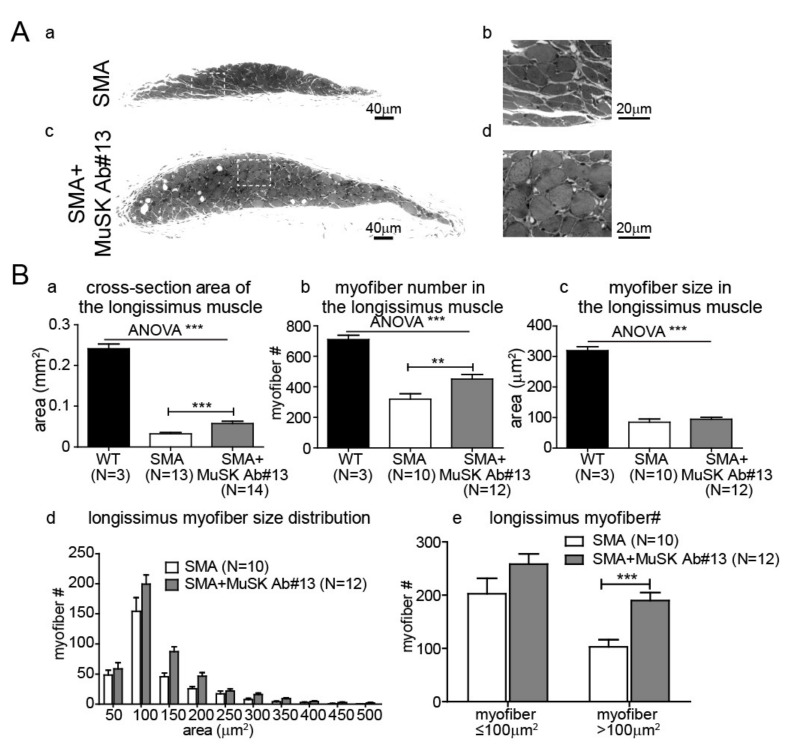
MuSK agonist antibody#13 increases the size of longissimus muscle in SMA mice. (**A**) Images of cross-sections of PND14 SMA longissimus muscles without (**a**,**b**)—white inset box in (**a**) is enlarged in (**b**)—and with (**c**,**d**)—white inset box in (**c**) is enlarged in (**d**)—MuSK agonist antibody #13 treatments. Scale bars: 40 µm in (**a**,**c**); 20µm in (**b**,**d**). (**B**) Quantitation of the cross-sectional area (**a**), myofiber number (**b**), and size (**c**) of the longissimus muscle in the PND 14 longissimus muscle of WT, delta7 SMA mice without and with MuSK agonist antibody #13 treatments. The distributions of myofiber size (**d**) and number of myofiber less and greater than 100 µm^2^ (**e**) of PND 14 delta7 SMA mice are also shown. Values are expressed as mean ± SEM, ** *p* < 0.01 and *** *p* < 0.001.

To further study the effects of MuSK antibody #13, we examined whether the antibody could reduce muscle atrophy in delta7 mice. As shown in Figure 4A,B, the splenius muscle of delta7 mice was severely atrophied, which is likely due to reduced functional innervation as previously shown [22]. Given that MuSK antibody #13 improves functional innervation in the vulnerable muscles, we first analyzed cross-sections of the splenius muscle by histology. Figure 4(Aa–d) shows that MuSK antibody #13 reduced muscle atrophy of the splenius muscle in delta7 mice. We quantitated muscle size, which showed that the increase in muscle cross-sectional area (Figure 4(Ba)) was attributed to an increase in myofiber number (Figure 4(Bb)), as well as the cross-sectional area of individual myofibers (Figure 4(Bc)). Furthermore, the distribution of individual myofiber sizes (Figure 4(Bd)) indicated that the increase in muscle size was primarily due to an increase in the number of large myofibers, especially those >100 µm^2^ in cross-sectional area (Figure 4(Be)). Similar enhancements in muscle size and myofiber number were also seen in another vulnerable muscle, the longissimus, following treatment of delta7 mice with MuSK antibody #13 (Figure 5).

**Figure 6:**
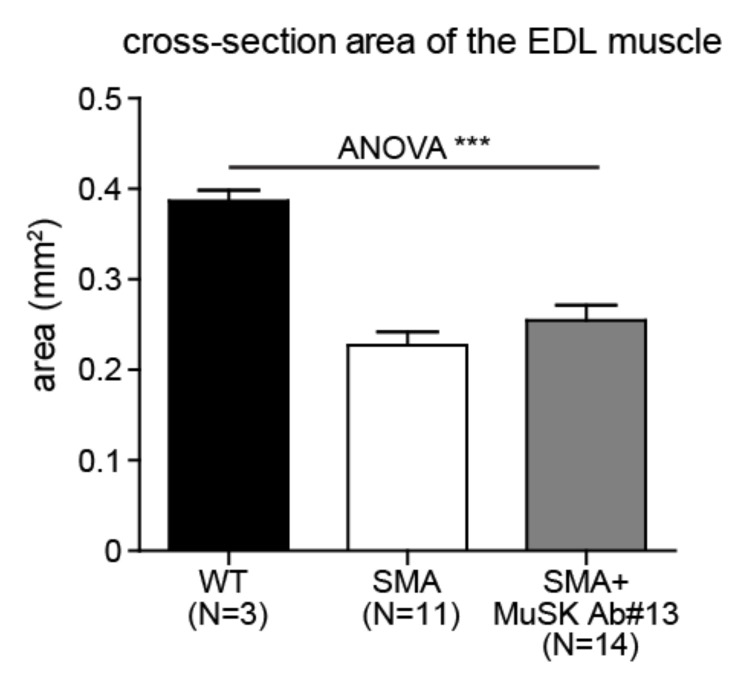
MuSK agonist antibody#13 does not affect the size of the EDL muscle in SMA mice. Quantitation of the cross-sectional area of the EDL muscle of PND14 WT and delta 7 SMA mice without and with MuSK agonist antibody #13 treatments. *** *p* < 0.001.

To determine whether the effect of MuSK antibody #13 on vulnerable muscles is primarily due to increased innervation, we also examined muscle morphometry in the resistant muscles, such as the extensor digitorum longus (EDL) muscle, which is fully innervated at end stage of delta7 mice [15,18]. As shown in Figure 6, MuSK antibody #13 did not alter the cross-sectional area of the EDL muscle, which is atrophied in delta7 mice as compared with WT mice. Thus, MuSK antibody #13 did not have a direct effect on muscle size or myofiber number, at least in the EDL muscle, indicating that the increase in muscle size and myofiber number in vulnerable muscles is likely due to improved NMJ innervation.

### 2.5. The EFFECT of MuSK Agonist Antibody #13 on Body Weight in SMA Mice

**Figure 7:**
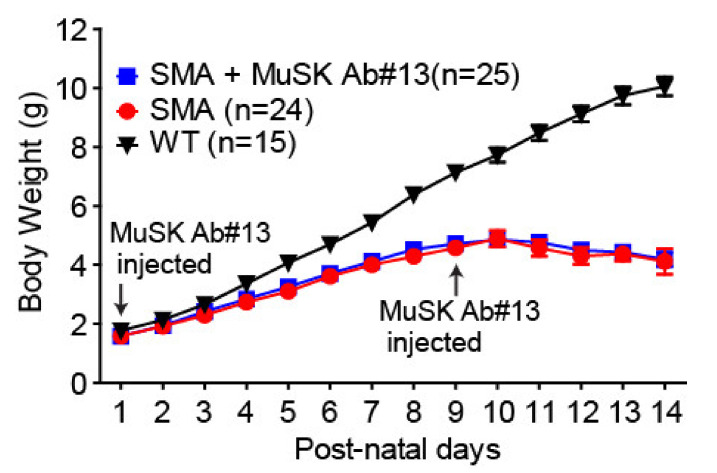
MuSK agonist antibody#13 does not affect the body weight of SMA mice. Quantitation of the body weight of WT and SMA mice between PND1 and PND14.

Given that MuSK antibody #13 increased the size of vulnerable but not resistant muscles, we did not anticipate that the antibody would enhance the body weight of delta7 mice. We measured the body weights of delta7 mice treated or untreated with MuSK antibody #13 from PND1 through PND 14 and found no significant increase in the body weight of the delta7 mice treated with MuSK antibody #13 (Figure 7). Thus, although MuSK antibody #13 attenuates NMJ defects and muscle atrophy in the vulnerable muscle of delta7 mice, the antibody did not by itself increase body weight and would not be expected to prolong life span.

## 3. Discussion

The SMA community has witnessed three recent breakthrough gene-targeted treatments for SMA patients: Spinraza (nusinersen), a SMN2-splicing antisense oligonucleotide approved by the US Food and Drug Administration (FDA) in 2016; Zolgensma (onasemnogene abeparvovec), an AAV9-mediated gene replacement therapy in 2019; and Evrysdi (risdiplam), a SMN2 splicing modifier small molecule in 2020 (see recent reviews by [43,44,45]). All of these three drugs significantly enhance the production of SMN protein levels and show remarkable efficacy in treating SMA patients. However, the clinical outcomes vary, and some patients show only a stabilization in motor function and continued appearance of neuromuscular symptoms [41,45,46,47]. The spectrum of clinical responses could be explained, in part, by initiation of treatment after symptoms were evident and at different stages of disease, especially for older children and adult patients. A well-established concept is that early drug treatment is critical for optimal motor function and clinical benefit in both SMA mice and SMA patients [9,48]. Treatment with SMN-enhancing drugs, once motoneurons have degenerated, is likely to provide limited benefit. Furthermore, while SMA affects primarily spinal motor neuron survival, recent studies have shown that SMA is a multisystem disease, including dysfunction in peripheral tissues and various organs [40,49,50,51,52]. Therefore, there remains a need to further identify and characterize novel therapeutic targets for SMA to complement existing SMN-enhancing drugs to provide maximal effectiveness across the life course for all subtypes of SMA patients [42].

One of the hallmarks of SMA are NMJ defects, which contribute to the major SMA disease symptoms in both animal models and SMA patients (see reviews by [8,46]). We have previously shown that SMN-modifiers and the FDA-approved Risdiplam improve NMJ innervation significantly, but even with the optimal treatments, NMJ deficits remain in some denervation-vulnerable muscles in the delta7 SMA mice [37,38]. Thus, improving NMJ innervation and function has the potential to complement the efficacy of SMN-enhancing drugs. Our work shows that MuSK agonist antibody #13 improved innervation and synaptic function of NMJs in SMA mice. These findings, in denervation-vulnerable muscles of SMA mice, are similar to earlier reports showing that increasing agrin expression improved innervation in tricep [29] and quadricep [30] muscles in delta7 mice. Recent studies, using MuSK agonist antibody #13, have shown an increase in NMJ innervation in the SOD1G93A mouse model of ALS [35,36]. Thus, enhancing synaptic differentiation by boosting MuSK activity has the potential to overcome or mitigate nerve terminal withdrawal in NMJ diseases, possibly by activating a retrograde signaling pathway to enhance motor nerve terminal attachment [35]. It would be interesting to examine whether the retrograde signaling through MuSK antibody #13 treatment could also prevent motor neuron loss to support one current strand of thinking that motor neuron death in SMA is attributed to “dying back” of motor nerves [13]. However, we showed that improving the NMJ innervation with MuSK antibody #13 occurred mainly in denervation-vulnerable muscles, such as the splenius muscle and the longissimus muscle (Figure 2). It is technically challenging to identify the motor neurons that innervate specially these denervation-vulnerable muscles in the delta7 SMA mice.

In addition to enhancing innervation, our work demonstrates a near doubling in size of vulnerable muscles, such as splenius or longissimus muscles. This increase in muscle size is primarily due to an increase in myofiber number. These findings suggest that improving innervation diminishes denervation-induced myofiber degeneration, rather than due to a direct effect on myofiber numbers and muscle size in these vulnerable muscles by the antibody treatment. These ideas are further supported by the failure of MuSK antibody #13 to increase muscle size in denervation-resistant muscles, such as the EDL (Figure 6), in which no significant denervation is observed in SMA mice [18]. These findings differ from those reported for boosting agrin expression, which results in an increase in myofiber size even in denervation-resistant muscles of SMA mice [29,30]. Given that the vast majority of muscles belong to resistant muscles in the delta7 mouse model of SMA, it is not surprising that there was no observed significant change in body weight in SMA mice treated with the MuSK agonist antibody. MuSK agonist antibody #13 also failed to increase body weight in the SOD1G93A mouse model of ALS [36].

This proof-of-principle study demonstrates that activation of MuSK using a MuSK agonist antibody reduces NMJ denervation, accompanied by improved synaptic efficacy and increased size of denervation-vulnerable muscles in delta7 model mice. Although this MuSK agonist antibody #13 would not alone function as therapy for SMA, a MuSK agonist antibody may complement other therapies for SMA by promoting synapse maintenance and preventing synapse disruption, thereby mitigating further deterioration of NMJ innervation and function, and maximizing therapeutic effectiveness for all types of SMA patients.

## 4. Materials and Methods

Animals: All animal experiments were conducted under Institutional Animal Care and Use Committee-approved protocols (Protocol Number: 11136-CR005, approved on 15 December 2016) at AAALAC certified animal facility. Delta7 mice were generated from breeder pairs of heterozygous delta7 mice purchased from the Jackson Laboratory (no. 005025, FVB.Cg-Tg(SMN2*delta7)4299AhmbTg(SMN2)89Ahmb Smn1tm1Msd/J). Genotypes were determined as described previously [15]. Transgenic mice carrying homozygous mouse Smn alleles were used as wildtype (WT) controls.

Drug treatments: MuSK agonist antibody #13 (provided by Genentech, [34]) was administered to wildtype and delta7 mice at postnatal day (PND)1 and PND9 via intraperitoneal (IP) injection at 10 mg/kg in saline solution.

Mouse histology: Animals were euthanized and perfused with Ringer’s solution (in mM, 135 NaCl, 5 KCl, 1 MgSO_4_, 15 NaHCO_3_, 1 Na_2_HPO_4_, 11 D-glucose, 2.5 Calcium gluconate, pH 7.4) followed by 4% paraformaldehyde. Tissues were stored at 4° in phosphate-buffered saline. To examine NMJ innervation, muscles were dissected and teased into thin layers of ~10 fibers. Nerve terminals were labelled with antibodies against neurofilament (1:2000; Millipore, Burlington, MA, USA) and vesicular acetylcholine transporter (vAChT, Synaptic Systems, Gottingen, Germany; 1:1000), and AChRs were labelled with α-bungarotoxin (Invitrogen, Waltham, MA, USA; 1:200). Innervations were quantified as described previously [18]. To assess muscle size, muscles were fixed with glutaraldehyde, followed by osmium tetroxide and embedded in Epon. Transverse sections at one micron were stained with toluidine blue, and the size of muscle cross-sectional area was measured using NIH Image software.

Electrophysiological recordings from NMJs: Conventional intracellular recording was performed as described previously [15]. The splenius muscle was dissected, and muscle contraction was blocked with 30 min incubation of 2–3 mM µ-conotoxin (Biomol, Swampscott, MA, USA) in oxygenated Ringer’s solution. Evoked endplate potentials (EPPs) were elicited by 1 Hz train stimulation in toxin-free Ringer’s solution.

Statistical analysis: Unequal variance two-tailed Student’s t-tests with statistic software (prism 5.0) were used to determine statistical difference and significance between delta 7. One-way analysis of variance (ANOVA) was performed to evaluate statistical difference among all groups. *p* < 0.05 was considered as significant (*** *p* < 0.001, ** *p* < 0.01 and * *p* < 0.05; ns, *p* > 0.05). Values were expressed as mean ± SEM.

## Data Availability

Data is contained within the article.

## References

[1] Sumner C.J., Paushkin S., Ko C.P. (2017). Spinal Muscular Atrophy: Disease Mechanisms and Therapy.

[2] Lefebvre S., Burglen L., Reboullet S., Clermont O., Burlet P., Viollet L., Benichou B., Cruaud C., Millasseau P., Zeviani M. (1995). Identification and characterization of a spinal muscular atrophy-determining gene. Cell.

[3] Lorson C.L., Hahnen E., Androphy E.J., Wirth B. (1999). A single nucleotide in the SMN gene regulates splicing and is responsible for spinal muscular atrophy. Proc. Natl. Acad. Sci. USA.

[4] Monani U.R., Lorson C.L., Parsons D.W., Prior T.W., Androphy E.J., Burghes A.H., McPherson J.D. (1999). A single nucleotide difference that alters splicing patterns distinguishes the SMA gene SMN1 from the copy gene SMN2. Hum. Mol. Genet..

[5] Feldkotter M., Schwarzer V., Wirth R., Wienker T.F., Wirth B. (2002). Quantitative analyses of SMN1 and SMN2 based on real-time lightCycler PCR: Fast and highly reliable carrier testing and prediction of severity of spinal muscular atrophy. Am. J. Hum. Genet..

[6] Crawford T.O., Pardo C.A. (1996). The neurobiology of childhood spinal muscular atrophy. Neurobiol. Dis..

[7] Sugarman E.A., Nagan N., Zhu H., Akmaev V.R., Zhou Z., Rohlfs E.M., Flynn K., Hendrickson B.C., Scholl T., Sirko-Osadsa D.A. (2012). Pan-ethnic carrier screening and prenatal diagnosis for spinal muscular atrophy: Clinical laboratory analysis of >72,400 specimens. Eur. J. Hum. Genet..

[8] Boyd P., Gillingwater T.H., Sumner C.J., Paushkin S., Ko C.P. (2017). Axonal and neuromuscular junction pathology in spinal muscular atrophy. Spinal Muscular Atrophy: Disease Mechanisms and Therapy.

[9] Kong L., Valdivia D.O., Simon C.M., Hassinan C.W., Delestree N., Ramos D.M., Park J.H., Pilato C.M., Xu X., Crowder M. (2021). Impaired prenatal motor axon development necessitates early therapeutic intervention in severe SMA. Sci. Transl. Med..

[10] Cifuentes-Diaz C., Nicole S., Velasco M.E., Borra-Cebrian C., Panozzo C., Frugier T., Millet G., Roblot N., Joshi V., Melki J. (2002). Neurofilament accumulation at the motor endplate and lack of axonal sprouting in a spinal muscular atrophy mouse model. Hum. Mol. Genet..

[11] Le T.T., Pham L.T., Butchbach M.E., Zhang H.L., Monani U.R., Coovert D.D., Gavrilina T.O., Xing L., Bassell G.J., Burghes A.H. (2005). SMNDelta7, the major product of the centromeric survival motor neuron (SMN2) gene, extends survival in mice with spinal muscular atrophy and associates with full-length SMN. Hum. Mol. Genet..

[12] Kariya S., Park G.H., Maeno-Hikichi Y., Leykekhman O., Lutz C., Arkovitz M.S., Landmesser L.T., Monani U.R. (2008). Reduced SMN protein impairs maturation of the neuromuscular junctions in mouse models of spinal muscular atrophy. Hum. Mol. Genet..

[13] Murray L.M., Comley L.H., Thomson D., Parkinson N., Talbot K., Gillingwater T.H. (2008). Selective vulnerability of motor neurons and dissociation of pre-and post-synaptic pathology at the neuromuscular junction in mouse models of spinal muscular atrophy. Hum. Mol. Genet..

[14] Kong L., Wang X., Choe D.W., Polley M., Burnett B.G., Bosch-Marce M., Griffin J.W., Rich M.M., Sumner C.J. (2009). Impaired synaptic vesicle release and immaturity of neuromuscular junctions in spinal muscular atrophy mice. J. Neurosci..

[15] Ling K.K., Lin M.Y., Zingg B., Feng Z., Ko C.P. (2010). Synaptic defects in the spinal and neuromuscular circuitry in a mouse model of spinal muscular atrophy. PLoS ONE.

[16] Ruiz R., Casanas J.J., Torres-Benito L., Cano R., Tabares L. (2010). Altered intracellular Ca^2+^ homeostasis in nerve terminals of severe spinal muscular atrophy mice. J. Neurosci..

[17] Dachs E., Hereu M., Piedrafita L., Casanovas A., Caldero J., Esquerda J.E. (2011). Defective neuromuscular junction organization and postnatal myogenesis in mice with severe spinal muscular atrophy. J. Neuropathol. Exp. Neurol..

[18] Ling K.K., Gibbs R.M., Feng Z., Ko C.P. (2012). Severe neuromuscular denervation of clinically relevant muscles in a mouse model of spinal muscular atrophy. Hum. Mol. Genet..

[19] Genabai N.K., Ahmad S., Zhang Z., Jiang X., Gabaldon C.A., Gangwani L. (2015). Genetic inhibition of JNK3 ameliorates spinal muscular atrophy. Hum. Mol. Genet..

[20] Valsecchi V., Boido M., De Amicis E., Piras A., Vercelli A. (2015). Expression of Muscle-Specific MiRNA 206 in the Progression of Disease in a Murine SMA Model. PLoS ONE.

[21] Schellino R., Boido M., Borsello T., Vercelli A. (2018). Pharmacological c-Jun NH2-Terminal Kinase (JNK) Pathway Inhibition Reduces Severity of Spinal Muscular Atrophy Disease in Mice. Front. Mol. Neurosci..

[22] Zhao X., Feng Z., Ling K.K., Mollin A., Sheedy J., Yeh S., Petruska J., Narasimhan J., Dakka A., Welch E.M. (2016). Pharmacokinetics, pharmacodynamics, and efficacy of a small-molecule SMN2 splicing modifier in mouse models of spinal muscular atrophy. Hum. Mol. Genet..

[23] Weatherbee S.D., Anderson K.V., Niswander L.A. (2006). LDL-receptor-related protein 4 is crucial for formation of the neuromuscular junction. Development.

[24] Kim N., Stiegler A.L., Cameron T.O., Hallock P.T., Gomez A.M., Huang J.H., Hubbard S.R., Dustin M.L., Burden S.J. (2008). Lrp4 is a receptor for Agrin and forms a complex with MuSK. Cell.

[25] Zhang B., Luo S., Wang Q., Suzuki T., Xiong W.C., Mei L. (2008). LRP4 serves as a coreceptor of agrin. Neuron.

[26] Yumoto N., Kim N., Burden S.J. (2012). Lrp4 is a retrograde signal for presynaptic differentiation at neuromuscular synapses. Nature.

[27] Okada K., Inoue A., Okada M., Murata Y., Kakuta S., Jigami T., Kubo S., Shiraishi H., Eguchi K., Motomura M. (2006). The muscle protein Dok-7 is essential for neuromuscular synaptogenesis. Science.

[28] Zhang Z., Pinto A.M., Wan L., Wang W., Berg M.G., Oliva I., Singh L.N., Dengler C., Wei Z., Dreyfuss G. (2013). Dysregulation of synaptogenesis genes antecedes motor neuron pathology in spinal muscular atrophy. Proc. Natl. Acad. Sci. USA.

[29] Kim J.K., Caine C., Awano T., Herbst R., Monani U.R. (2017). Motor neuronal repletion of the NMJ organizer, Agrin, modulates the severity of the spinal muscular atrophy disease phenotype in model mice. Hum. Mol. Genet..

[30] Boido M., De Amicis E., Valsecchi V., Trevisan M., Ala U., Ruegg M.A., Hettwer S., Vercelli A. (2018). Increasing Agrin Function Antagonizes Muscle Atrophy and Motor Impairment in Spinal Muscular Atrophy. Front. Cell Neurosci..

[31] Kaifer K.A., Villalon E., Smith C.E., Simon M.E., Marquez J., Hopkins A.E., Morcos T.I., Lorson C.L. (2020). AAV9-DOK7 gene therapy reduces disease severity in Smn(2B/-) SMA model mice. Biochem. Biophys. Res. Commun..

[32] DeChiara T.M., Bowen D.C., Valenzuela D.M., Simmons M.V., Poueymirou W.T., Thomas S., Kinetz E., Compton D.L., Rojas E., Park J.S. (1996). The receptor tyrosine kinase MuSK is required for neuromuscular junction formation in vivo. Cell.

[33] Perez-Garcia M.J., Burden S.J. (2012). Increasing MuSK activity delays denervation and improves motor function in ALS mice. Cell Rep..

[34] Xie M.H., Yuan J., Adams C., Gurney A. (1997). Direct demonstration of MuSK involvement in acetylcholine receptor clustering through identification of agonist ScFv. Nat. Biotechnol..

[35] Cantor S., Zhang W., Delestree N., Remedio L., Mentis G.Z., Burden S.J. (2018). Preserving neuromuscular synapses in ALS by stimulating MuSK with a therapeutic agonist antibody. Elife.

[36] Sengupta-Ghosh A., Dominguez S.L., Xie L., Barck K.H., Jiang Z., Earr T., Imperio J., Phu L., Budayeva H.G., Kirkpatrick D.S. (2019). Muscle specific kinase (MuSK) activation preserves neuromuscular junctions in the diaphragm but is not sufficient to provide a functional benefit in the SOD1(G93A) mouse model of ALS. Neurobiol. Dis..

[37] Ratni H., Ebeling M., Baird J., Bendels S., Bylund J., Chen K.S., Denk N., Feng Z., Green L., Guerard M. (2018). Discovery of Risdiplam, a Selective Survival of Motor Neuron-2(SMN2) Gene Splicing Modifier for the Treatment of Spinal Muscular Atrophy (SMA). J. Med. Chem..

[38] Naryshkin N.A., Weetall M., Dakka A., Narasimhan J., Zhao X., Feng Z., Ling K.K., Karp G.M., Qi H., Woll M.G. (2014). Motor neuron disease. SMN2 splicing modifiers improve motor function and longevity in mice with spinal muscular atrophy. Science.

[39] Feng Z., Ling K.K., Zhao X., Zhou C., Karp G., Welch E.M., Naryshkin N., Ratni H., Chen K.S., Metzger F. (2016). Pharmacologically induced mouse model of adult spinal muscular atrophy to evaluate effectiveness of therapeutics after disease onset. Hum. Mol. Genet..

[40] Groen E.J.N., Talbot K., Gillingwater T.H. (2018). Advances in therapy for spinal muscular atrophy: Promises and challenges. Nat. Rev. Neurol..

[41] Darrow J.J., Sharma M., Shroff M., Wagner A.K. (2020). Efficacy and costs of spinal muscular atrophy drugs. Sci. Transl. Med..

[42] Hensel N., Kubinski S., Claus P. (2020). The Need for SMN-Independent Treatments of Spinal Muscular Atrophy (SMA) to Complement SMN-Enhancing Drugs. Front. Neurol..

[43] Chen T.H. (2020). New and Developing Therapies in Spinal Muscular Atrophy: From Genotype to Phenotype to Treatment and Where Do We Stand?. Int. J. Mol. Sci..

[44] Ojala K.S., Reedich E.J., DiDonato C.J., Meriney S.D. (2021). In Search of a Cure: The Development of Therapeutics to Alter the Progression of Spinal Muscular Atrophy. Brain Sci..

[45] Ravi B., Chan-Cortes M.H., Sumner C.J. (2021). Gene-Targeting Therapeutics for Neurological Disease: Lessons Learned from Spinal Muscular Atrophy. Annu. Rev. Med..

[46] Mercuri E., Pera M.C., Scoto M., Finkel R., Muntoni F. (2020). Spinal muscular atrophy-insights and challenges in the treatment era. Nat. Rev. Neurol..

[47] Gollapalli K., Kim J.K., Monani U.R. (2021). Emerging concepts underlying selective neuromuscular dysfunction in infantile-onset spinal muscular atrophy. Neural. Regen. Res..

[48] Monani U.R., Osborne M.A., Lutz C., Sumner C.J., Paushkin S., Ko C.P. (2017). Temporal requirements for the survival motor neuron protein. Spinal Muscular Atrophy: Disease Mechanisms and Therapy.

[49] Hamilton G., Gillingwater T.H. (2013). Spinal muscular atrophy: Going beyond the motor neuron. Trends Mol. Med..

[50] Shababi M., Lorson C.L., Rudnik-Schoneborn S.S. (2014). Spinal muscular atrophy: A motor neuron disorder or a multi-organ disease?. J. Anat..

[51] Nash L.A., Burns J.K., Chardon J.W., Kothary R., Parks R.J. (2016). Spinal Muscular Atrophy: More than a Disease of Motor Neurons?. Curr. Mol. Med..

[52] Yeo C.J.J., Simeone S.D., Townsend E.L., Zhang R.Z., Swoboda K.J. (2020). Prospective Cohort Study of Nusinersen Treatment in Adults with Spinal Muscular Atrophy. J. Neuromuscul. Dis..

